# Illness and disease: an empirical-ethical viewpoint

**DOI:** 10.1186/s12910-018-0341-y

**Published:** 2019-01-09

**Authors:** Anna-Henrikje Seidlein, Sabine Salloch

**Affiliations:** Institute of Ethics and History of Medicine, University Medicine Greifswald, Ellernholzstr. 1-2, 17487 Greifswald, Germany

**Keywords:** Concept of disease, Illness, Sickness, Subjective views, Patient beliefs, Empirical ethics, First-person experience

## Abstract

**Background:**

The concepts of disease, illness and sickness capture fundamentally different aspects of phenomena related to human ailments and healthcare. The philosophy and theory of medicine are making manifold efforts to capture the essence and normative implications of these concepts. In parallel, socio-empirical studies on patients’ understanding of their situation have yielded a comprehensive body of knowledge regarding subjective perspectives on health-related statuses. Although both scientific fields provide varied valuable insights, they have not been strongly linked to each other. Therefore, the article aims to scrutinise the normative-ethical implications of patient perspectives in building a bridge to the empirical ethics debates.

**Main text:**

Three potential fields of tension between the illness and the disease perspective are presented. Consequently, findings from empirical research examining patient perspectives on illness are displayed and the practical implications and associated ethical issues which arise are discussed. This leads to the conclusion that an explicit and elaborate empirical-ethical methodology is needed to deal appropriately with the complex interaction between patients’ views and the medico-professional view of disease. Kon’s four-stage model of normative-empirical collaboration is then applied against the background of empirical data on patient perceptions. Starting from this exemplary approach, the article suggests employing empirical-ethical frameworks for further research on the conceptual and normative issues, as they help to integrate perspectives from the philosophy of medicine with socio-empirical research.

**Conclusion:**

The combination of theoretical and empirical perspectives suggested contributes to a more nuanced discussion of the normative impact of patients’ actual understanding of illness. Further empirical research in this area would profit from explicitly considering potential ethical issues to avoid naturalistic fallacies or crypto-normative conclusions that may compromise healthcare practice. Vice versa, medico-theoretical debates could be enriched by integrating subjective views of those people who are immediately affected.

## Background

The concept of illness and related ideas such as disease and sickness have developed into a complete “network of medical concepts” ([1], p. 8). Similar to other terms, such as injury, malady and disability, the ways in which they are used sometimes seem arbitrary and interchangeable in everyday language. By contrast, the terms have been the subject of scientific controversy for a long time and still remain without a “standard, normative meaning” ([2], p., 360). The conceptual triad of disease, illness and sickness – first introduced by Twaddle in 1968 [3] – has been especially widespread within this discourse. Twaddle defines disease as “physiological malfunction […] independent of subjective experience and social conventions” ([4], pp. 8-9). In Fleischman’s words, it simply “removes the patient from the pathology” ([5], p. 7). Conversely, illness refers to a “subjectively interpreted undesirable state of health” ([4], p. 10). Sickness, finally, stands for “a social identity […] defined by others with reference to the social activity of that individual” ([4], p. 11).

Twaddle’s conceptual triad is used nowadays in various scientific disciplines, such as sociology or philosophy of medicine, and has undergone extensive theoretical examination (e.g. [6]). The discussion generated multiple efforts to strengthen rigor, overcome deficiencies and to reconceptualise the triple division or its elements. Nordenfelt has especially criticised the conceptual division, as it does not allow any conclusions as long as it is not well-founded in a comprehensive theory of health [7, 8]. Sadegh-Zadeh summarises critically that the only result after decades of debate consists of “a few labels” ([9], p., 605) that transgress logical reasoning and do not allow a clear assignment of specific conditions to disease and illness as they are “all a matter of degree” ([9], p., 607). He, therefore, strives towards a new definition of these elements based on the idea of “fuzziness”.

The emphasis and application of the three concepts in western societies, whose medical paradigm is based on the triple distinction to a great degree, can be regarded as either a bridge or a barrier between the perspectives [7]. The former emphasis promotes the importance of lay views and prompts us to listen to the patients’ stories and feelings [10, 11] in times of medicalisation (e.g. [12, 13]) of live events and experiences. The latter accentuation stresses that referring to the distinction between the perspectives can lead to discrimination and “widens the gap between what patients seek and doctors provide” ([14], p. 9). Furthermore, it suggests that there is something like an objective “professional or expert” view of disease in contrast to the subjective illness experience of “lay” people (e.g. [2]). Danner Clouser et al., for example, highlight that the disease perspective presents itself as “more robust ontologically” ([15], p. 29). Unlike illness, disease seems to allow an objective assessment and detection of reasons with scientific methods that are expected to reveal an ultimate truth.

**Fig. 1:**
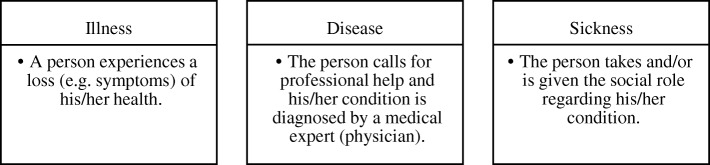
Interrelatedness of the triad according to Nordenfelt’s “standard *medical encounter*” [17], own illustration

Boyd [16] holds that it is “elusive” to arrive at a consensus definition of disease, illness and sickness. Nevertheless, it can be noticed as a broad agreement and shared common understanding that the three concepts emphasise different aspects inherent in human ailment. As such, they can serve as a valuable heuristic to explore and analyse phenomena that appear in healthcare practice. One prominent interpretation of the interrelatedness between illness, disease and sickness (the “paramount example”) was suggested by Nordenfelt [17]. According to his position, the subjective experience of illness leads to seeking help from a medical expert who diagnoses the disease. This expert labelling then has consequences regarding the social (sickness) role of the person affected (Fig. 1).

The interpretation and usage of the concepts of disease, illness and sickness have a considerable impact on various facets of health care and social life. Within the medical system, for example, the labelling of an individual as having a disease often remains contested: there are certain statuses, such as “internet addiction” [18–20], “burn out” or “obesity” [21], which are – not least depending on the specific national and cultural context – considered as disease or not. In addition, clinicians might disagree whether a person is actually affected by a certain disease or whether the limits of normalcy have not been surpassed in the concrete case. The ascription of having a disease becomes more complicated by the fact that the medically accepted thresholds are subject to constant modifications, as the recent case of US guidelines on hypertension shows impressively [22, 23].

Regarding illness, the patients’ concrete experiences have a considerable influence on health-related behaviour, adherence and the willingness to cooperate with healthcare personnel and institutions, as psychological research has shown [24–29]. Furthermore, the subjectively experienced burden of disease is, in the end, crucial for the success of diagnostic or therapeutic measures. The immediate experience of illness, however, does not usually appear in a non-reflective way. Instead, the way patients deal with illness is accompanied by the development of subjective explanatory models, for example regarding causes and modifying factors. The term “illness” (as used in this article), thus, comprises immediate and reflected appreciation of the ailment from the patients’ point of view. One can also include the patients’ engagement with the medico-professional perspective on disease in the latter, which may shape their bodily and mental perceptions as well as their explanatory models.

The concept of sickness, finally, which refers to the social and societal dimensions of ailment, has consequences for such different fields as the sick role in family contexts, health insurance and compensation or working place conditions. The “sickness dimension” of being ill or having a disease, thus, makes us aware that medical conditions cannot be reduced to biomedical or biostatistical conditions or to the subjective feelings of those who are affected. Instead, it is deeply embedded in social practices, ethical traditions and legal systems.

Such practical implications of the concepts of disease, illness and sickness for individuals’ lives and social practice are widely uncontested. At the same time, however, the concrete scope and normative impact of health-related concepts and their interrelatedness are still under discussion. The greatest part of the more recent debates in the philosophy of medicine focuses on the concept of disease, its theoretical embedding and normative implications [30–34]. Attempts to classify the various understandings of disease are extremely varied and do not allow an unambiguous and definite systematisation, for example, along the lines of naturalist and non-naturalist approaches [35–37]. However, in addition to the interpretation of disease, the concrete content of the illness concept is equally important from a scientific and practical or normative standpoint. Nevertheless, it has not attracted equal attention in the medico-theoretical debates so far. Consequently, this paper focuses on the duo of illness and disease, seeking to further clarify their interrelatedness beyond Boorse’s observation that they “are related somewhat as are low intelligence and stupidity” ([38], p. 84). Due to the complexity of the field, the concept of sickness will not be considered explicitly in this article. Many ideas developed in the paper (particularly regarding empirical research), however, are also highly relevant concerning sickness and related concepts such as the sickness role or sickness absence.

Starting from the observation that subjective interpretations of health have relevant consequences for individual people, the healthcare system and society, the article aims to further clarify the normative-ethical implications of patient perspectives. Insights from the debates in the philosophy of medicine will be confronted with interdisciplinary *empirical* studies on actual patient perspectives, which illuminate the narratives of those who are affected. In referring to debates on empirical ethics, this article aims to reconcile perspectives from the philosophy of medicine with socio-empirical research to arrive at a more nuanced discussion of the normative impact of patients’ actual understanding of illness. This can help researchers from different disciplines conducting empirical studies on illness perspectives to align their work better with the normative discourse. In addition, it can support healthcare professionals to foster learning from the “*moral pathography*” [39] presented and, thus, make sense of clinical experiences they have in therapeutic relationships.

## Main text

Medico-theoretical discussions on disease and illness operate mostly independently of a second branch of research which is, however, closely related in terms of content: socio-empirical studies on patients’ actual understanding of their health-related situation. Whereas the – epistemic and normative – importance of patient perspectives is generally acknowledged [40], explicit links between the empirical research and medico-theoretical debates are mainly missing.

A large variety of methodological approaches has already been applied to study patient experiences of illness: qualitative research designs using in-depth interviews (generating narratives of illness experience) or focus groups and quantitative surveys measuring attitudes and mixed-method approaches appear [41–43]. In addition, researchers have used different forms of art, such as poems and paintings, as data sources [44]. The following section provides a brief overview of the research areas selected exemplarily examining patients’ experiences and perspectives on illness. The study selection is guided by three potential fields of tension between the illness and the disease perspective: (1) divergent interpretations of causes and context of the same condition, (2) illness in the absence of disease, and (3) disease in the absence of illness. Each of the three sections will highlight practical consequences and ethical challenges arising from the deviation between the empirically documented illness and the medico-professional perspective on disease.

### Field of tension I: Illness and disease in contrast

Patients’ understanding of illness is typically presented in the form of narratives and, therefore, qualitative research designs are often used. Such research, on the one hand, aims to illuminate the “lived experience” of illness, focusing especially on what it means to live with a certain condition, for example, mental disorder [45], Huntington’s [46], Parkinson’s [47] or gastro-oesophageal reflux [48]. On the other hand, patients’ subjective views are explored to understand how they conceptualise their illness regarding circumstances and underlying causes [49–51]. The conceptions of illness particularly of patients living with a mental disorder have been investigated. Kangas [52], for example, conducted research on theories of depression through narratives of individuals suffering from this condition. Patients described different beliefs regarding reasons why they became depressed: shortcomings of childhood, work-related burn-out and provoking factors, such as life events associated with intense emotions leading to depression. Peter et al. [53] conducted in-depth interviews with individuals diagnosed with different mental health problems and reported how these patients’ perception of disease changed throughout the therapeutic experience.

Concerning somatic conditions, a difference has been demonstrated between patients suffering from chronic symptoms and those experiencing an acute onset: whereas the acutely ill often try to regain normalcy after disruption due to their impairment [54], the chronically ill describe their illness as omnipresent and as a never-ending work [55, 56] that forces them to adjust in all aspects of life and makes planning impossible [57–59]. In addition, culture has been shown to be a major factor that shapes beliefs, influences help-seeking behaviour, decision-making concerning treatment and the treatment expectations and outcomes (e.g. [60]). Maier and Straub, who did research on traumatised migrants state that: “There is a high potential for misunderstandings, and obviously a large gap between the respective concepts of illness and appropriate treatment” ([61], p., 233). Public health policies and prevention campaigns are particularly likely to fail if they do not take socio-cultural backgrounds into account [62].

It has also been demonstrated that patients’ theories regarding the cause and treatment of their illness, which may contrast with biomedical evidence and scientific ideas of aetiology, have an impact on health-related behaviour [27, 63–65]. The expectations patients have of certain treatments vary widely depending on their different perceptions of illness. This also has an impact on what kind of therapy they are willing to accept and adhere with. Studies have proved the significance of views of illness not only regarding coping and health-related behaviour, such as self-care, therapeutic adherence [66] and secondary prevention, but also rehabilitation (physical health) or recovery (mental health) and self-stigma.

Ethical challenges may arise if perceptions of illness counteract medico-professional definitions of disease and its prevention, e.g. regarding cancer [67]. Patients’ theories might be refuted as lay beliefs and, therefore, be regarded as irrational. In addition, ethical concerns arise in dealing with traditional methods of healing and the question when and how these attempts should be stopped because of expected negative consequences for the patients or their surroundings [68]. If these and other deviations between subjective interpretations and the medico-professional perspective on disease remain undetected, this can have a negative impact on prevention, therapy, rehabilitation and recovery as well as on the patient-physician relationship. Normative questions, thus, arise about ways to acknowledge both perspectives appropriately aiming at a fair integration of illness narratives with the scientific state-of-the-art.

### Field of tension II: Illness without disease

The examples described so far refer to patients diagnosed with a certain disease who are feeling ill; at the same time, however, their concrete illness experience and explanatory models differ from the medico-professional perspective. Subjective illness and medically acknowledged disease, nonetheless, do not inevitably occur coincidentally. Instead, people may feel ill without professionals detecting a disease. This pertains, for example, to undesirable bodily complaints, such as pain, that is generally understood as a symptom leading the physician to the diagnose of a disease but sometimes appears without any physical cause [69]. Some of these symptoms (such as pain or fatigue) and syndromes (such as irritable bowel syndrome or fibromyalgia) remain without an obvious somatic cause, even after extensive diagnosis and testing. Thus, they are often referred to as “medically unexplained symptoms” (MUS) [70, 71]. Another example within in this field of tension includes cases where a physical disease has already been cured from a medical perspective but the person affected still feels ill [72]. Additionally, some patients see the doctor with complaints which make them feel ill (weakness, slowness and other indicators for frailty) but that can be explained by age-related changes in bodily functions and loss of functional capacity and, therefore, are not acknowledged as a disease by most physicians [73].

In the situations described, professionals often perceive patients as complicated and feel overstrained when they frequently reappear asking for help. Physicians also might become unsure how to react to patients’ suffering when no accurate (physical) diagnosis can be made. As a result, these patients are sometimes interpreted as having a psychosomatic character, which leads to a range of communicative and ethical challenges [71, 74].

From an ethical perspective, it is important to further analyse whether people who feel ill should be treated as healthy if there are no measurable, objective parameters to detect a disease. This is, for example, relevant regarding (long-term) sickness absence caused by MUS. Other challenges arise concerning obtaining the patient’s informed consent, which might be difficult or even unfeasible if it remains uncertain which diagnostic procedures and therapeutic options will reduce the patient’s burden of symptoms. Balancing the risk-benefit ratio to determine the further course of action in MUS cases is complicated but the abandonment of such an evaluation can cause serious harm through (missing) surgery, medication or diagnostic imaging [75]. Finally, how to address MUS in the communication process and decide what degree of certainty about the absence of a (rare) disease is necessary remains a major challenge.

Patients suffering from illness without medically detected disease do not only challenge the contemporary paradigm and culture of medicine, which prioritises medical and scientific explanations, but also strain healthcare resources [76] – for example, when patients insist on MRI and other costly diagnostic procedures – so that matters of justice in health care are affected when the perception of illness deviates from the medical perspective on diagnostics and treatment [71].

### Field of tension III: Disease without illness

A third phenomenon regarding potential tensions between disease and illness relates to people who have been diagnosed with a certain disease but who are not feeling ill. The topic is closely linked to the impact of medico-technological advances, for example, routinely screening for specific diseases such as cancer. In this case, a disease is often detected at a very early stage when people are symptomless and do not feel ill. Martinez [77], for example, analysed the ambiguous experiences of women diagnosed with different forms and stages of cervical cancer (precursors) through using the Papanicolau smear test. These women describe themselves as being simultaneously in a state of subjective, health because they do not feel pain or other restrictions. Martinez summarises the women’s situation as “living on the borderlands of health, disease, and illness” (p. 798) where the diagnosis creates feelings of “disembodiment” (p. 800). Similar experiences of “struggling with unreliability of body” ([78], p. E446) are described in men diagnosed with prostate cancer and individuals with screening-detected colorectal cancer diagnosis [79] who assess themselves as being healthy.

Furthermore, this field of tension includes constellations where patients deny the existence of the attested disease. Some people, for example, claim HIV (human immunodeficiency virus) to be harmless and not to cause AIDS (acquired immunodeficiency syndrome) or any other serious diseases and still other denialism movements doubt that HIV exists at all [80]. Other groups of patients reject the credibility of scientific explanatory models concerning the origin of AIDS and build conspiracy theories [81]. Antiretroviral therapy, for them, is seen only as an instrument to increase the pharmaceutical industry’s profits. As a result of both denialism and conspiracy beliefs, prevention of HIV transmission and therapy of AIDS are significantly hindered.

Practical implications of the examples mentioned above, where individuals are diagnosed with a disease but do not feel ill, are manifold. Foremost, for example, regarding prostate cancer, the confusing situation where objective medical data and subjective feelings of well-being are contradictory, makes it hard for the people affected to choose between different treatment options, such as active surveillance or initial aggressive treatment, for example, surgery [82, 83]. Furthermore, in cases where the risk-benefit ratio is borderline or controversial, this conflict aggravates, as sometimes it is not even sure whether the individual will ever suffer from symptoms or become seriously harmed because of the disease diagnosed [84]. Instead, individuals might experience impairment of physical and mental well-being due to diagnostic procedures, the diagnosis they receive and unnecessary treatment. Long-term effects may mean that individuals are scared of having a disease because they experience that not suffering from symptoms is not necessarily an indicator for health. Consequently, people can become confused and uncertain about their body perception and might lose trust in their health-related feelings.

Ethical questions in this field arise when asymptomatic patients are diagnosed with a certain state that is treated as a disease, leaving their subjective perspective of feeling healthy unappreciated. Such an overemphasis of disease towards illness can lead to overdiagnosis [12, 85, 86], which is closely related to the sovereignty to define the patient’s condition. Although many individuals who are overdiagnosed in screening tests highlight the benefit (rather than the harm) which they perceive due to the feeling that they owe their lives to the overdiagnosis (known as the “popularity paradox” [87–89]), this cannot fully neutralise a patient’s right to an interpretative priority regarding their state of health. If the patient feels that his or her perception is not taken seriously, the physician-patient relationship can be compromised, resulting, for example, in a loss of trust. The concept of epistemic injustice, more specifically in this case, a “*credibility deficit*” [90] called “testimonial injustice” [90, 91], aims to capture this phenomenon which has been applied to analyse the medical field by Kidd and Carel [92–94].

There are further ethical challenges inherent to the appropriateness of medical care and the question of how to find the balance between over- and undertreatment especially regarding children [95, 96], mental illness [97] and culturally shaped beliefs. Furthermore, divergent perspectives concerning the state of health can lead to the question how to proceed with people who do not accept their diagnosis and refuse treatment. Informed consent can be challenging or impossible when patients do not feel ill at all, as one of the major preconditions is undermined: if the person affected does not appreciate the medical data introduced by the physician (or is not willing to accept them, respectively), consent to further diagnostic or treatment measures does not occur on an informed basis.

### Evaluating the normative impact of patient perspectives on disease

The brief overview of selected fields of empirical research investigating patient perspectives on their conditions has demonstrated the variety of ethical questions which arise concerning the potential tension between illness as a subjective perception and disease as a medico-scientific attribution. Such normative issues, however, are currently not well reflected in the empirical research on patient experiences. The practical use which can legitimately be made from empirical knowledge about patient perspectives depends highly on normative premises regarding the authoritative status attributed to such findings. The transition from empirical findings on patients’ illness experiences to normative claims regarding the health care provided should be carefully reflected against the background of ethical theories, concepts or principles to avoid is-ought fallacies and related problems [98].

Empirical-ethical research has emerged as an innovative research field in the last two decades and is dedicated explicitly to the integration of socio-empirical data and normative theories, principles or concepts. Manifold positions can be taken principally regarding the interaction between the normative and descriptive aspects which are included in ethical judgements. Classifications of the empirical-ethical domain have been suggested using such criteria as the distinction between descriptive and prescriptive science, the locus of moral authority, types of normativity used and more [99]. Specific questions arise, for example, regarding the role of empirical data in the regulation of health care and biotechnology [100] or the adaptation of quality criteria for empirical-ethical research [101]. In addition to such theoretical reflections, various concrete methodologies have been suggested for conducting empirical-ethical studies in biomedicine and health care [102]. Making explicit the normative-empirical interaction in concrete research projects helps to unveil crypto-normative premises which often underlie practical conclusions drawn from empirical data [103].

Recent reviews demonstrate that the proportion of empirical publications in bioethics continues to increase [104] and that most European bioethicists are using empirical methods in their work [105]. Empirical-ethical studies deal with a wide range of bioethical topics, such as compulsory treatment in psychiatry [106], genetic testing [107], assisted reproduction [108] and the end-of-life context [109]. The topic of what constitutes a disease, however, has only rarely been addressed in the empirical-ethical debates [110].

An explicit link between the broad field of socio-empirical studies on patients’ perceptions of illness and the empirical-ethical debate is, thus, greatly lacking. A comprehensive and explicit reflection on the normative-empirical interaction, however, would be desirable for empirical studies on patient perspectives, for example, regarding the acknowledgement of patients’ explanatory models and health-related behaviour in the physician-patient relationship and their implications for the wider contexts of healthcare institutions and policies. Divergent empirical-ethical frameworks could be applied potentially to this field to make the relationship between empirical data and normative questions, principles and arguments explicit. In the following section, a suggestion will be made to utilise Kon’s differentiation between “Lay of the Land”, “Ideal Versus Reality”, “Improving Care” and “Changing Ethical Norms” to clarify the normative impact of patient perspectives on illness.

### Four stages of normative-empirical collaboration

In a 2009 article, Alexander A. Kon exhibits four categories to classify empirical research in bioethics, which illuminate the interaction between descriptive data and normative-ethical issues [111]. Whereas Kon considers the four categories as being equally important and helpful, he, nevertheless, describes a hierarchical order between them: the scientific work of the higher categories builds logically on those insights derived in the lower categories. Kon exemplifies the four categories and their logical coherence by using empirical studies on patient autonomy. They can, however, also be read against the background of empirical data on patient perspectives on illness. Kon’s classification represents a rather pragmatic approach to arranging and handling the complex field of empirical-ethical research. In this, however, it can help researchers who conduct socio-empirical research on patients’ perception of illness to further clarify issues of normative-empirical interaction in their studies. It is, therefore, taken in the remainder of this article to sketch some first suggestions of how such a classification of empirical-ethical research could contribute to the quality of empirical research on patient viewpoints, particularly regarding the normative impact of the “illness perspective”.

Kon’s first category, “Lay of the Land”, aims to “define current practices, opinions, beliefs, or other aspects that may be considered the status quo” [111]. Such descriptive or explanatory work can not only provide starting points for further research but may also reveal perspectives for improving care. Concerning patient perspectives on disease, “Lay of the Land” can, for example, reveal differences between illness conceptions of differently affected groups (e.g. adults and children) or between the medico-scientific notion of disease and patients’ subjective perspectives on illness. Whereas normative questions are not answered directly by this unveiling work, it may still be helpful to explain problems occurring in clinical practice. Furthermore, ethical perspectives are opened and explicitly acknowledged regarding the authoritative status of (e.g. psychiatric) patient perspectives, which deviate from the “standard” view of disease in the professional context.

Building on “Lay of the Land” studies, “Ideal Versus Reality” research (Kon’s second category) assesses the extent to which clinical practice reflects ethical norms. Such studies are usually hypothesis-driven and aim for changes in the healthcare system. Research on patients’ illness perceptions may fulfil this “Ideal Versus Reality” function, for example, regarding the practice of informed consent in minors. In many countries, physicians are legally required to involve children in medical decision-making to an extent which is appropriate to the child’s developmental status and capacity. This requirement may also include respect towards children’s conception of their own illness – even if it deviates from the view of parents or healthcare professionals. Empirical knowledge about children’s actual understanding may, thus, be helpful to assess how far the norm to appropriately involve children in decision-making is valid and could be taken as a basis to restructure clinical practice.

The third category, called “Improving Care”, refers to projects which design and test novel methods aiming at ensuring compliance with ethical norms. So far, there are few (or no) empirical studies related to patient perspectives on illness which evaluate interventions for the improvement of clinical care. However, new research designs could be developed in this field. Healthcare institutions, for example, could implement communication training which explicitly includes aspects such as the patients’ own explanatory models for their condition and their interpretation of saluto- or pathogenetic factors. Patient satisfaction or therapy adherence could, for example, serve as endpoints for an evaluation of these programmes. In this way, the dealing deliberately with subjective conceptions of illness could be used for the further development of a patient-centred health care.

Finally, Kon’s category of “Changing Ethical Norms” is designated to studies and comprehensive analyses in which empirical findings inform ethical principles. This process may lead to an adaption of ethical norms regarding aspects that particularly count in practice. Kon uses the example of the development of our understanding of shared decision-making which has emerged, inter alia, from an over-emphasis on autonomous choice, which is not mirrored in practice. Regarding patient perspectives on illness, “Changing Ethical Norms” could mean that public health policies might deviate from medico-technical explanations of disease if this increases their acceptance in a population which adheres, for example, to animist or natural medicine. It might be helpful for the sake of prevention or to improve the access to healthcare services to also include the perspectives of those who are affected, even if they do not match medical science.

## Conclusions

The brief analysis of prominent research fields and their normative impact demonstrates that further research on patient perspectives on illness would profit from explicitly considering potential ethical issues emanating from the results. These issues should be discussed against the background of empirical-ethical frameworks to deal systematically with the normative significance of patient perspectives on illness. A distinct empirical-ethical approach can help to avoid is-ought fallacies and crypto-normative conclusions that may arise from empirical studies on patient perspectives and compromise health-care practice. Designing studies in this way could also enrich the debate on empirical ethics methodology, as it allows a special focus on issues such as lay versus professional perspectives, epistemic injustice and the authenticity of viewpoints which are elicited in socio-empirical (mainly qualitative) research.

The medico-theoretical debates on disease, illness and sickness, on the other hand, could profit from a better consideration of the actual subjective views of those people who are experienced with illness. For example, the complex relationship between patients’ immediate experience of symptoms and their reflective dealing with illness and the professional perspective on disease can be understood more realistically when considering the results of empirical research. A fuller and differentiated understanding of patients’ actual experiences could enrich concepts from the philosophy of medicine regarding certain practice fields (e.g. the building of empirically based middle-range theories).

Further theoretical questions refer to the normative status of patient perspectives generally and to methodological issues of how to document the authentic voices of those who are primarily affected [40]. The increasing importance particularly of patient and public involvement makes us aware of the necessity of respecting patient perspectives in an appropriate form and preventing them being instrumentalised for the purposes of distinct groups. Theoretically reflected empirical research on patient perspectives of disease can help us in this and other aims and contribute to the ethical quality of health care.

## Abbreviations

AIDS: acquired immunodeficiency syndrome
HIV: human immunodeficiency virus
MRI: magnetic resonance imaging
MUS: medically unexplained symptoms
